# Is Persistent Ketamine Use a Valid Model of the Cognitive and Oculomotor Deficits in Schizophrenia?

**DOI:** 10.1016/j.biopsych.2008.10.045

**Published:** 2009-06-15

**Authors:** Celia J.A. Morgan, Vyv Huddy, Michelle Lipton, H. Valerie Curran, Eileen M. Joyce

**Affiliations:** a Clinical Psychopharmacology Unit, University College London, United Kingdom; b Division of Neuroscience and Psychological Medicine, Imperial College London, United Kingdom; c Institute of Neurology, University College London, United Kingdom

**Keywords:** Antisaccades, drug abuse, eye tracking, ketamine, schizophrenia, smooth pursuit, spatial working memory, verbal memory

## Abstract

**Background:**

Acute ketamine has been shown to model features of schizophrenia such as psychotic symptoms, cognitive deficits and smooth pursuit eye movement dysfunction. There have been suggestions that chronic ketamine may also produce an analogue of the disorder. In this study, we investigated the effect of persistent recreational ketamine use on tests of episodic and working memory and on oculomotor tasks of smooth pursuit and pro- and antisaccades.

**Methods:**

Twenty ketamine users were compared with 1) 20 first-episode schizophrenia patients, 2) 17 polydrug control subjects who did not use ketamine but were matched to the ketamine users for other drug use, and 3) 20 non-drug-using control subjects. All groups were matched for estimated premorbid IQ.

**Results:**

Ketamine users made more antisaccade errors than both control groups but did not differ from patients. Ketamine users performed better than schizophrenia patients on smooth pursuit, antisaccade metrics, and both memory tasks but did not differ from control groups.

**Conclusions:**

Problems inhibiting reflexive eye movements may be a consequence of repeated ketamine self-administration. The absence of any other oculomotor or cognitive deficit present in schizophrenia suggests that chronic self-administration of ketamine may not be a good model of these aspects of the disorder.

Acute administration of ketamine in healthy volunteers elicits psychotic symptoms and cognitive deficits similar to those seen in schizophrenia (1–3). Recreational ketamine use is associated with psychotic symptoms such as delusions and episodic memory impairment, also seen in schizophrenia (4). Chronic ketamine administration has been proposed as a more representative model of cognitive impairments seen in schizophrenia (5). People who persistently self-administer ketamine provide a naturalistic opportunity to investigate whether there are similarities in the behaviors disrupted in schizophrenia and following chronic *N-*methyl-D-aspartate (NMDA) receptor antagonism.

In addition to cognitive impairment, schizophrenia is associated with oculomotor deficits, particularly in smooth pursuit (6) and the ability to inhibit reflexive eye movements when performing the antisaccade task (7). Three previous studies have found abnormal smooth pursuit following an acute dose of ketamine (8–10), but there have been no reports of oculomotor function following persistent ketamine use.

In the current study, we wished to examine whether persistent ketamine use is associated with oculomotor and cognitive deficits typical of schizophrenia. We compared recreational ketamine users with IQ- and age-matched early-phase schizophrenia patients. We also tested a healthy control group and a group to control for the effect of nonketamine substance use. We hypothesized that similar abnormalities would be observed in persistent ketamine users and schizophrenia patients.

## Methods and Materials

### Participants

Twenty ketamine users were recruited from a database and via a snowball sampling technique (11). Because the ketamine users also used other drugs, 17 polydrug users who did not use ketamine but who were matched with the ketamine group for other recreational drug use were also recruited. Inclusion criteria for the ketamine group were 1) age between 18 and 50, 2) ketamine use for at least 1 year and at least twice per month, 3) ketamine use within the last month but not in the last 3 days (verified by urinanalysis), and 4) absence of current or past history of mental health problems.

Twenty patients with schizophrenia and 20 healthy control subjects were selected for comparison on the basis of age and National Adult Reading Test IQ (12) from a prospective, longitudinal study of first-episode psychosis in West London. Inclusion criteria and clinical assessments have previously been described in detail (13). Briefly, patients had presented with a psychotic illness for the first time and received no more than 12 weeks of antipsychotic medication. All 20 patients included in this study met DSM-IV diagnostic criteria for schizophrenia. Eighteen were taking antipsychotic medication (15 of whom were taking second-generation medications). Twenty healthy control subjects were recruited from the same catchment area as the patients; exclusion criteria were a past or family history of psychiatric illness, previous head injury, or other illness affecting brain function and drug or alcohol abuse or dependence.

The local Research Ethics Committees approved the study. All participants gave written informed consent and reimbursed for their time.

### Oculomotor Tasks

#### Apparatus

Eye movements were recorded with an SR-Research (Mississauga, Canada) Eyelink eye tracker. Three cameras simultaneously recorded the position of both eyes and the head, allowing gaze position to be computed without head restraint. Participants viewed a 21-inch monitor from a distance of 60 cm, which subtended a visual angle of 40 horizontally and 30 vertically.

#### Smooth Pursuit

Stimuli consisted of a red circle presented against a black background. The circle subtended approximately .5 of visual angle and, starting on the left, moved horizontally backward and forward ± 27. Two blocks of six cycles of constant velocity pursuit were generated. Each block was preceded by a drift correct procedure that lasted approximately 3 sec. In the first block, the target moved at a constant velocity of 5 of visual angle per second; in the second block this increased to 10 per second, and in the third block, this further increased to 20 per second.

#### Prosaccade and Antisaccade Tasks

Each trial comprised the following sequence: 1) a central fixation dot appeared at the beginning of each trial; 2) after 800 msec, the central dot disappeared, a peripheral target dot appeared simultaneously for 1000 msec, and a 200-msec click signal sounded. For prosaccade trials, subjects were asked to direct their gaze as quickly and accurately as possible to the new dot and then return to the central fixation point. For antisaccade trials, subjects were instructed to move their eyes rapidly toward an equidistant position in space, but in the opposite direction to the peripheral stimulus, that is, to the mirror-image location. There were 24 trials for each condition.

### Neuropsychology

#### The Rey Auditory Verbal Learning (RAVLT) (14)

In trials 1–5, subjects were read the same list of 15 nouns and asked to recall immediately afterward as many as possible. A second interference list was then read followed by a free recall test. Delayed free recall for the original list was tested, without reminding, after a short and long (30-min) delay, followed by a test of recognition memory.

#### Spatial Working Memory (15)

Patients were required to “open” sets of boxes, varying between three and eight in number, to find tokens. Errors were recorded when boxes in which tokens had been found were reopened; a measure of strategy was also taken.

### Data Analysis

#### Pursuit Task

Eye movements were analyzed using custom software written in LabVIEW. The software extracted eye position data consisting of a series of x axis pixel coordinates sampled every 2 msec. Sections of pursuit that included intrusive saccades or corrective saccades, blinks, or fixations were not included in the analysis. Within each operator-defined period of pursuit, the program calculated the velocity gain (ratio of eye velocity to target velocity), which provides an accurate measure of the ability of the smooth pursuit system to match eye velocity to target velocity (16).

#### Prosaccade and Antisaccade Tasks

The detection of saccades was based on a velocity criterion of 30°/sec in addition to acceleration across three consecutive samples. In both paradigms, spatial accuracy measures of primary saccade gain (saccadic amplitude divided by target amplitude) and final eye position (the longest stable period of fixation after any corrective saccades had been made, expressed as gain) as well as latency to perform a correct antisaccades (milliseconds) were calculated. In the antisaccade task, the number of errors when subjects made an initial saccade toward the target was recorded.

### Statistical Analysis

Data were analyzed with SPSS Version 11.5. Repeated-measures analysis of variance (ANOVA) was used to compare the groups for the smooth pursuit data, with Group as a between-subjects factor. Other data were analyzed with one-way ANOVAs; Kruskall-Wallis tests were used when data were nonparametrically distributed. When significant differences emerged, Bonferroni-corrected planned comparisons were conducted: ketamine versus nondrug, ketamine versus polydrug, and ketamine versus schizophrenia.

## Results

### Participants

See Table 1. There were no significant differences in estimated premorbid IQ or age at which subjects left full-time education. There was a significant difference in sex, reflecting the absence of any female subjects in the polydrug using group. There was a nonsignificant trend for an age difference between the groups. When we examined the impact of age or sex on our findings, neither significantly affected the outcome of the analyses.

### Drug Use

Ketamine was used for 2.7 (± 1.07) days/month over 3.23 (± 1.26) years with 1.92 (± .86) g/session. There were no differences between ketamine and polydrug control groups in the number of subjects taking other drugs regularly (cocaine: 8/20 vs. 5/17; cannabis: 11/20 vs. 8/17; ecstasy: 9/20 vs. 10/17). Smoking and alcohol use data were not recorded for the nondrug control subjects, but there were no significant differences between the other three groups in numbers of smokers or regular drinkers.

### Smooth Pursuit

See Table 2. Repeated-measures ANOVA of velocity gain revealed a main effect of Target Speed and Group. Performance worsened with increasing speed for all groups; schizophrenia patients were worse than ketamine users (*p* = .020).

### Prosaccades

See Table 2. There were no significant differences between the groups.

### Antisaccades

**Figure 1:**
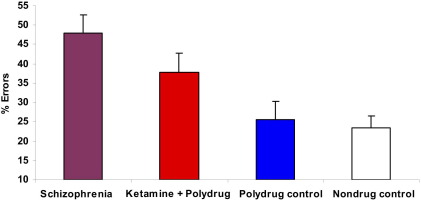
Antisaccade errors (bars represent standard error). Significant differences (*p* < .05) were observed between the ketamine group and both the polydrug and non-drug-using control groups.

Groups differed in the number of antisaccade errors [Figure 1; *F*(3,73) = 4.34, *p* = .006] and oculomotor metrics (Table 2). Ketamine users demonstrated more errors than nondrug (*p* = .025) and polydrug (*p* = .048) control subjects but not schizophrenia patients. For correct trials, schizophrenia patients were worse than ketamine users for antisaccade latency (*p* < .001), final eye position (*p* = .041), and primary saccade gain (*p* = .003).

### Neuropsychology

See Table 3. There were group differences for all measures except spatial working memory strategy. Ketamine users were better than schizophrenia patients on verbal learning and showed fewer spatial working memory errors (*p* < .05).

## Discussion

Compared with matched groups of nonketamine polydrug users and non-drug-using control subjects, ketamine users made significantly more antisaccade errors but were no different on spatial accuracy and latency of antisaccades or on accuracy of smooth pursuit. Compared with schizophrenia patients, ketamine users were no different with respect to antisaccade errors but performed better on all other indices of oculomotor control; they also performed better on tests of verbal learning and spatial working memory. Although the patients were taking antipsychotic medication, we have previously shown that smooth pursuit and antisaccade performance is not impaired by treatment in early-phase schizophrenia (17,18), and other studies have found that similar cognitive impairment is present in untreated groups (19), suggesting that this is not an explanation of the worse performance of the patient group. Rather, our results indicate that persistent ketamine use may not be a good model of these aspects of schizophrenia, at least at the levels and frequency of intake described in this sample.

A possible explanation of the isolated increased antisaccade error rate, compared with other preserved antisaccade and oculomotor metrics, is that antisaccade errors relate to response suppression and indicate a nonspecific impairment of inhibitory control common to many types of addiction (20). However, antisaccade errors were at normal levels in a group of nonketamine drug users who were otherwise matched for the range and extent of other substances used, although reports of other drug use were reliant on self-report. Alternative explanations that cannot be distinguished in his study include the effect of persistent ketamine use on dopaminergic function or a preexisting trait that predisposed this group to take the drug.

## Figures and Tables

**Table 1:** Group Characteristics

	Ketamine Users	Schizophrenia	Polydrug-Using Control	Non-Drug-Using Control	*F*(3,73)	*p*
	(*n* = 20)	(*n* = 20)	(*n* = 17)	(*n* = 20)		
Age at Testing (range)	28.2 (20–42)	24.8 (17–48)	26.47 (18–30)	30.8 (21–51)	2.64	.06
NART IQ (range)	114.25 (93–126)	111.05 (105–120)	110.2 (92–122)	112.3 (105–121)	1.04	.38
Age Left Education (range)	18.85 (16–21)	17.7 (15–21)	17.94 (16–21)	18.5 (16–21)	1.36	.20
Sex (male/female)	15/5	14/6	17/0	11/9	9.91^a^	.02
Smokers (*n*)	12	14	8	Not recorded	4.25^a^	.119
Alcohol Users (*n*)	20	18	20	Not recorded	2.03^a^	.362

NART, National Adult Reading Test.

a χ^2^ statistic.

**Table 2:** Group Performance for Smooth Pursuit and Prosaccade and Antisaccade Metrics

	Ketamine Users (SD)	Schizophrenia Patients (SD)	Polydrug Control Subjects (SD)	Non-Drug-Using Control Subjects (SD)		
Smooth Pursuit					Main Effects and Interaction	
5° Velocity Gain	1.00 (.05)	.96 (.07)^a^	1.01 (.05)	1.00 (.04)	*Target Speed*	
					[*F*(2,149) = 27.12, *p* < .001]	
10° Velocity Gain	.99 (.06)	.96 (.07)^a^	1.01 (.06)	1.00 (.04)	*Group*	
					[F(3,70) = 4.06, *p* = .010]	
20° Velocity Gain	.95 (.11)	.87 (.11)^a^	.98 (.06)	.97 (.06)	Target Speed × Group	
					[*F*(6,140) = 1.67, *p* = .130]	
Prosaccades					*F*(3,73)	*p* Value
Latency (msec)	153.01 (21.50)	168.34 (56.50)	151.27 (21.59)	166.00 (19.01)	1.10	.355
Primary Saccade Gain	.94 (.06)	.92 (.06)	.94 (.05)	.96 (.06)	1.85	.150
Final Eye Position	1.0 (.01)	.93 (.20)	1.0 (.00)	1.0 (.02)	2.71^c^	.440
Antisaccades					*F*(3,73)	*p* Value
Latency (msec)	264.31 (39.80)	377.29 (99.70)^b^	267.31 (29.09)	282.29 (48.57)	14.58	<.001
Primary Saccade Gain	1.29 (.41)	.84 (.47)^b^	1.28 (.58)	1.02 (.53)	3.67	.016
Final Eye Position	1.25 (.34)	.97 (.25)^a^	1.36 (.56)	1.27 (.52)	10.91^c^	.012

Measures are mean (SD).

a < .05 for comparisons schizophrenia versus ketamine, polydrug versus ketamine, and control versus ketamine.

b < .01 for comparisons schizophrenia versus ketamine, polydrug versus ketamine, and control versus ketamine.

c Kruskall Wallis χ^2^ approximation for nonparametric data.

**Table 3:** Group Performance on the Rey Auditory Verbal Learning Test (RALVT) and Cambridge Automated Neuropsychological Testing Battery Spatial Working Memory Task (SWM)

	Ketamine Users	Schizophrenia Patients	Polydrug Control Subjects	Non-Drug-Using Control Subject	F*p*
RVLT Free Recall	48.95 (6.10)	43.25 (9.67)^a^	45.80 (6.80)	54.84 (5.48)^×^	9.164 <.001
RVLT Interference	10.18 (1.79)	8.35 (2.28)^b^	9.67 (2.61)	11.47 (2.12)	6.854 <.001
RVLT 30-min Free Recall	10.36 (2.32)	7.80 (2.65)	9.00 (2.14)	11.84 (2.61)	9.749 <.001
SWM Strategy Score	31.09 (5.63)	32.50 (8.13)	32.53 (6.42)	32.58 (4.49)	.275 .844
SWM Total Errors	15.68 (14.29)	30.40 (19.81)^b^	18.87 (15.59)	16.95 (13.06)	3.623 .017

Measures are mean (standard deviation). Differences from ketamine group in planned comparisons: schizophrenia versus ketamine, polydrug versus ketamine, control versus ketamine.

a Nonsignificant trend.

b *p* = .05.
